# Case report: Rare intrapulmonary malignant mesothelioma complicated with myositis

**DOI:** 10.3389/fonc.2022.1074821

**Published:** 2022-12-22

**Authors:** Dandan Gong, Jian Yu, Xiaodong Jiang

**Affiliations:** Department of Critical Care Medicine, the Second Hospital of Dalian Medical University, Dalian, Liaoning, China

**Keywords:** mesothelioma, cancer-associated myositis, oncology, lung parenchyma, primary

## Abstract

Malignant pleural mesothelioma is an uncommon aggressive tumor. Its incidence is even lower when the lung parenchyma is the primary site. Myositis is a common paraneoplastic syndrome, but it rarely presents with malignant pleural mesothelioma. This report presents a rare intrapulmonary malignant mesothelioma complicated by cancer-associated myositis. The patient presented with limb muscle weakness as the first symptom and was diagnosed with intrapulmonary malignant mesothelioma complicated by cancer-associated myositis on the basis of clinical, histological, immunohistochemical, and radiological findings. The patient responded poorly to conventional hormone therapy and died of respiratory failure within 2 months after the first presence of limb muscle weakness.

## 1 Introduction

Malignant mesothelioma is an aggressive tumor, associated with poor prognosis, which arises from the pleura, peritoneum, pericardium, or tunica vaginalis testis (1). Malignant pleural mesothelioma (MPM), which accounts for 90% of malignant mesothelioma cases (2), has proven to be a massive challenge for clinicians and scientists, with a median survival time of 9–12 months (3). MPM typically encases the lungs as a thick rind, and involvement of the lung parenchyma is rare. Here, we report a patient with myositis as a paraneoplastic syndrome with pulmonary parenchymal involvement.

## 2 Case description

A 66-year-old male presented to our hospital with a 1-month history of limb muscle weakness. He was an active smoker (20 cigarettes/day), had a family history of lung cancer. He had a history of diabetes mellitus for 15 years, no history of cancer and immune checkpoint inhibitors therapy.He also had no exposure history of asbestos.

In March 2022, the patient presented with limb muscle weakness and subcutaneous edema without any apparent cause. He was admitted to Zhongshan Hospital, affiliated with Dalian Medical University, for acute ileus in April 2022. Chest computed tomography (CT) performed during hospitalization showed a space-occupying lesion in the right upper lobe. The patient was treated conservatively and discharged.

**Figure 1:**
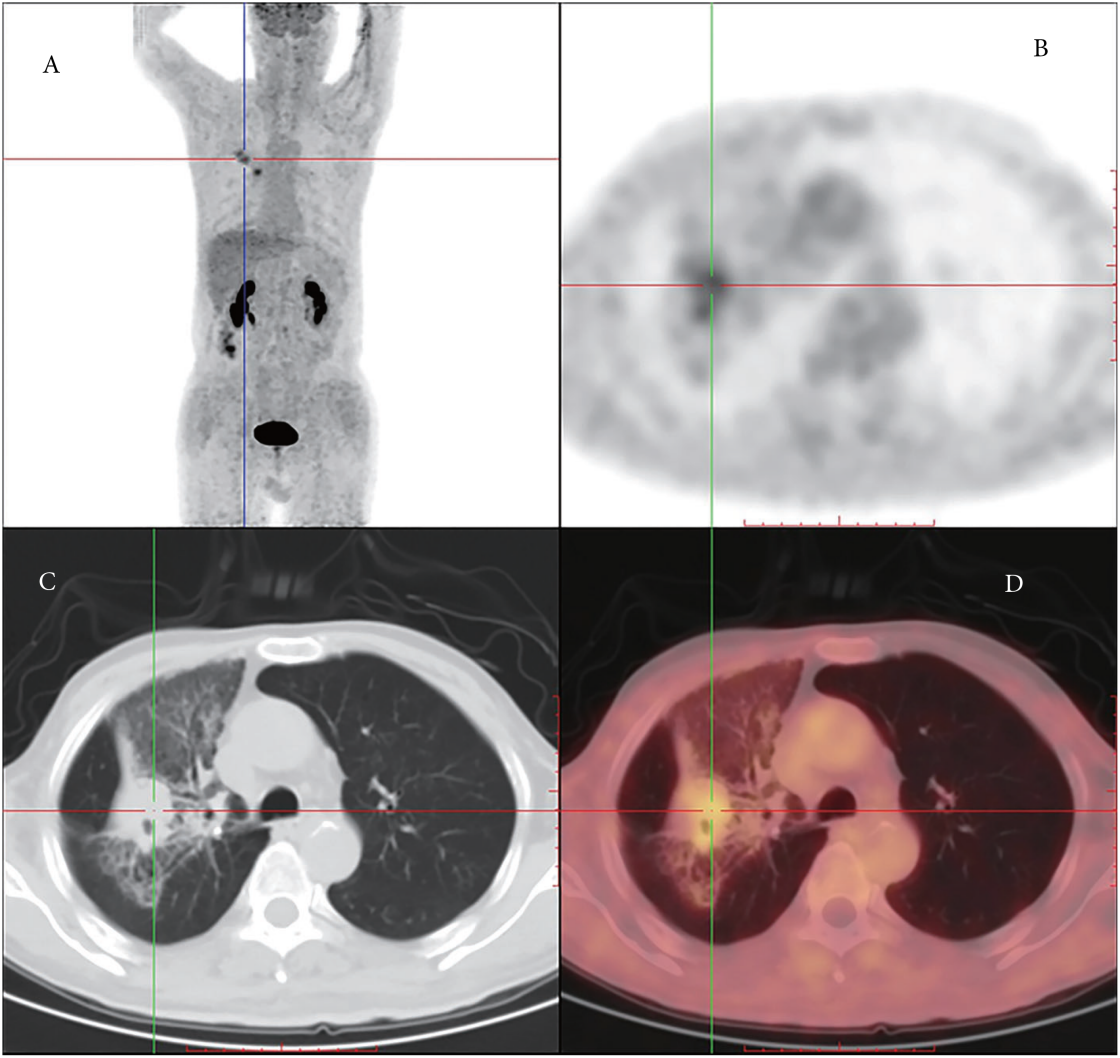
**(A)** Coronal PET (positron emission tomography) image shows higher FDG (fluorodeoxyglucose) uptake in the right lung and the lymph nodes on the left side. **(B)** Axial PET images. **(C)** Chest PET-CT presenting mesothelioma. **(D)** Axial PET images with higher tracer uptake.

Furthermore, positron emission tomography-CT was performed in our hospital, which revealed a soft tissue density occupying the lesion and enlarged lymph nodes in the right lobe, measuring 26 mm and 18 mm in diameter, respectively. No abnormal uptake was observed at other sites, including the lungs and pleura (**Figure 1**). Thus, the tumor was considered a primary tumor.

The patient was admitted to our hospital for diagnosis. After admission,he was presented with progressive limb muscle weakness (lower extremities:grade 1-2,upper extremities:grade 3-4), expectorating sputum hardly and respiratory muscle weakness.And he was transferred from inpatient unit to the intensive care unit (ICU) because of acute respiration failure complicating severe pneumonia at a week after admission.

Blood cell counts were within normal ranges upon admission, but the admission laboratory tests revealed definite abnormalities: high aspartate aminotransferase (AST, 536.09 U/L), alanine aminotransferase (ALT, 11.23 U/L), creatine kinase (CK; 12919.26 U/L), and creatine kinase (CK)-MB (CK-MB, 140.12 U/L) levels. Moreover, the level of CA125 increased to 182.73 U/mL (normal, <30.2 U/ml), CYFRA21-1 to 6.11 ng/mL (normal, <3.3 ng/mL), and NSE to 82.89 (normal, <16.3 ng/mL). Without hormone therapy, the levels of ALT (102.74 U/L), AST (157.65 U/L), CK (2768.34 U/L), and CK-MB (24.07U/L) became normal after 5 days.

**Figure 2:**
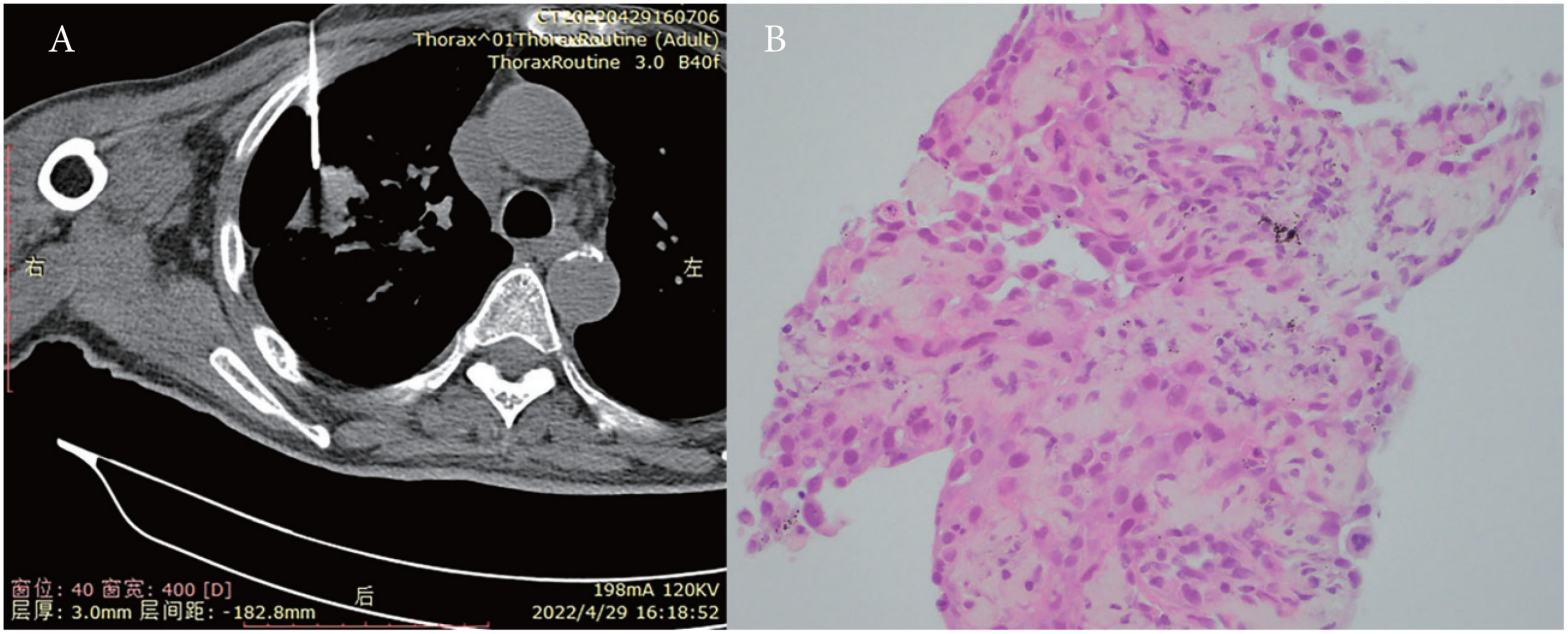
**(A)** Percutaneous biopsy of lung lesions guided by fusion CT. **(B)** Photomicroscopy with H&E (hematoxylin and eosin) staining presented unusual epithelioid cells.

**Figure 3:**
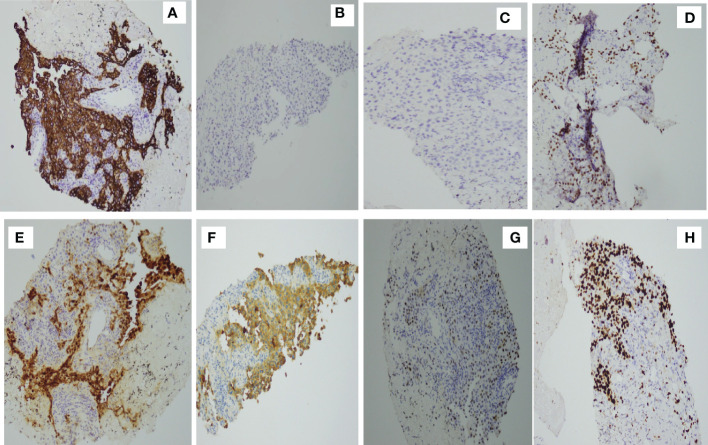
Immunohistochemical studies. Tumor cells are positive for **(A)** CK(+) **(D)** p63, **(E)** calretinin, **(F)** CK5/6, **(G)** WT-1, and **(H)** Ki-67 but negative for **(C)** p40 and **(B)** TTF-1.

Percutaneous biopsy of the lung lesions guided by fusion CT was performed after admission (**Figure 2**). Photomicroscopy with hematoxylin and eosin staining revealed unusual epithelioid cells of the primary tumor. Immunohistochemically, the tumor cells were positive for calretinin, CK, p36, CK5/6, WT-1, and Ki-67 but negative for TTF-1 and p40 (**Figure 3**). Sputum culture was performed and the detection of stenotrophomonas maltophilia, enterococcus faecium, and candida albicans in sputum of patients were postive.These results suggested intrapulmonary malignant mesothelioma complicated with pneumonia. As the poor performance status of the patient,surgery and radiotherapy were not suitable. Antibiotic therapy was performed with combination of cefepime,vancomycin,and caspofungin.

Cranial magnetic resonance imaging (MRI) and lumbar puncture were performed to investigate the potential causes of limb muscle weakness. However, there were no definite abnormalities in the symptoms of limb muscle weakness. Electromyography showed bilateral myogenic damage to the upper limbs, and nerve conduction tests revealed damage to the peripheral nervous system of all four limbs and delayed F-waves in the bilateral tibial nerve. Antibodies against nRNP/Sm, Sm, SSA, Ro52, SSB, Scl-70, Jo-1, and rRNP detected by enzyme-linked immunosorbent assay were all negative, whereas his auto-antibody profile showed anti-nuclear matrix protein 2 (NXP2) antibody positivity, which indicated cancer-associated myositis.

On the basis of these findings, we diagnosed the patient with intrapulmonary malignant mesothelioma complicated by myositis, which is the leading cause of limb muscle weakness. Upon diagnosis, the patient received 7 days of methylprednisolone (80 mg/day), but he had a poor response with progressive aggravation of limb muscle weakness and emergent expiratory dyspnea complicating pneumonia and died of respiratory failure. It was only 2 months from the first presence of limb muscle weakness onset to death.

## 3 Discussion

The 66-year-old patient presented with limb muscle weakness as the first symptom and was found a mass (2.6 cm) of the right upper lobe. He was an active smoker and had a family history of lung cancer, who had no history of asbestos exposure, cancer and immune checkpoint inhibitors therapy (ICI). According to the result of histological, immunohistochemical, and radiological findings,he was diagnosed as intrapulmonary malignant mesothelioma. Malignant mesothelioma often presents with diffuse involvement of the serous membranes, localized malignant mesotheliomas are extremely rare solitary circumscribed nodular tumors (4). Only very few cases have been reported that presented in ovary (5), liver (6), pancreatic (7) and lung (8). Diagnosing intrapulmonary malignant mesothelioma on the basis of clinical findings alone is difficult. A combination of histological, immunohistochemical, and radiological findings is needed. In our case,morphology and immunostains (positivity with calretinin,WT-1, and CK5/6; negativity with TTF-1 and p40) were consistent with an epithelioid malignant mesothelioma. For radiological investigations, European Society For Medical Oncology recommends applying CT scanning of the thorax for the localization of tumor sites and distant metastases or detecting early response to treatment. In our case,a soft tissue density occupying the lesion and enlarged lymph nodes in the right lobe were found and no abnormal uptake was observed at other sites.

Malignant mesothelioma are associated with risk factors including asbestos exposure, related minerals, simian virus 40, and nitrosamine (9). Asbestos exposure is the most important cause of malignant mesothelioma, accounting for 70–80% of cases (10). In the present case, the patient did not have any history of asbestos exposure, but he had a history of smoking and a family history of lung cancer.

Platinum-pemetrexed–based systemic therapy is a standard-of-care treatment in malignant mesothelioma (11). To early MPM,maximal tumor reduction surgery is recommended. While surgical resection for mesothelioma is always incomplete and it is not possible to cure someone with surgery alone, the multimodal approach to malignant pleural mesothelioma is gradually becoming the standard of care for MPM in patients with good performance status (12, 13). For inoperable patients, radiotherapy and chemotherapy was recommended. In recent year, immunotherapy and mesothelin-targeted therapies for treatment of patients with advanced unresectable mesothelioma has been highlighted (14). In our case, the patient was in a critical condition with acute respiration failure and severe pneumonia, who was not suitable for surgery, radiotherapy or chemotherapy.Antibiotic therapy and intensive care was performed.

Cancer-associated myositis is defined as the development of a malignancy within 3 years of the diagnosis of myositis, which is one of the typical paraneoplastic syndromes. Cancer-associated myositis have been increasingly referred as being related with a variety of malignancies, including hematological and solid cancers (15). While cancer-associated myositis rarely presents with MPM (16). In our case, there was no definite abnormality for symptoms of limb muscle weakness on cranial MRI and lumbar puncture. Antibodies to related immunological markers for the diagnosis of primary rheumatic diseases were all negative, whereas the auto-antibody profile showed NXP2 antibody positively.NXP2 antibodies are reportedly associated with cancer-associated myositis (17). Although, myasthenia gravis was reported to be induced by immune checkpoint inhibitors (ICI) (18), the patient in our case had no history of ICI therapy.The result of cranial MRI, lumbar puncture, and immunological profile were inferred that the myositis in the present case was cancer-associated. Methylprednisolone was used for treatment of the myositis. While the patient poorly responsed to methylprednisolone, after receiving 7 days of methylprednisolone, he died of respiratory failure.

## 4 Conclusion

In conclusion, cancer-associated myositis may be present in patients with intrapulmonary malignant mesothelioma. To our knowledge, this case, in which the patient experienced limb muscle weakness as the first symptom, is the first to report intrapulmonary malignant mesothelioma complicated with cancer-associated myositis. It was a fast-progression and poor-prognosis disease, and had a poor response to methylprednisolone.

## Data availability statement

The original contributions presented in the study are included in the article/supplementary material. Further inquiries can be directed to the corresponding author.

## Ethics statement

The studies involving human participants were reviewed and approved by The Ethics Committee of the Second Affiliated Hospital of Dalian Medical University. The patients/participants provided their written informed consent to participate in this study. Written informed consent was obtained from the individual(s) for the publication of any potentially identifiable images or data included in this article.

## Author contributions

DG, JY and XJ conceived the idea for the article. XJ drafted the manuscript. XJ approved the final version of the manuscript. All authors contributed to the article and approved the submitted version.
